# Rheological Control of Microsphere Expansion and Cellular Stabilization in Styrene–Butadiene–Styrene Foams

**DOI:** 10.3390/polym18212696

**Published:** 2026-10-31

**Authors:** Ahmet Hakan Cinar, Zeynep Keskin, Furkan Sarisoy, Gökçe Yilmaz, Zuhal Yurtbasi, Emine Kasgoz, Alper Kasgoz

**Affiliations:** 1 Polymer Materials Engineering Department, Faculty of Engineering, Yalova University, 77100 Yalova, Turkey; ahmethakancinar7815@gmail.com (A.H.C.); keskinzeynep274@gmail.com (Z.K.); furkansarisooyy@gmail.com (F.S.); gkurt945@gmail.com (G.Y.); zuhal.yurtbasi@yalova.edu.tr (Z.Y.); eminekasgoz@polyshield.com.tr (E.K.); 2 Polyshield Engineering Consultancy and R&D Co., 77100 Yalova, Turkey

**Keywords:** styrene–butadiene–styrene (SBS), thermally expandable microspheres, rheological control, cellular stabilization, lightweight foams

## Abstract

Lightweight elastomeric foams for footwear, sealing and damping applications require substantial density reduction without loss of cellular stability, yet the role of matrix rheology in expandable-microsphere foaming of oil-extended thermoplastic elastomers remains insufficiently understood. Herein, styrene–butadiene–styrene (SBS) foams were prepared with 1–3 phr thermally expandable microspheres, 10–30 phr additional processing oil and three nominal densities (0.80, 0.65 and 0.45 g cm^−3^). Increasing oil content reduced matrix elastic and flow resistance by approximately 2–3-fold and increased the mean pore size from 25.2 to 49.6 µm under the intermediate-density condition. Under the lowest-density condition, however, excessive softening reduced cellular stability, raising the pore-size coefficient of variation to 49.7% for LD-30-3. Combined optical microscopy and dielectric measurements showed that larger pores at comparable density did not necessarily correspond to a higher bulk void fraction, while the permittivity response was consistent with a predominantly discrete closed-cell structure. Greater pore expansion did not coincide with maximum mechanical performance: the highest mean toughness (11.50 MJ m^−3^) was obtained for HD-10-3. Cyclic testing showed that increasing oil content reduced load-bearing capacity but nearly doubled conditioned damping (from approximately 3–4% to 6–7%). Overall, matrix rheological resistance can be used to balance microsphere expansion, cellular retention and function-specific performance in lightweight SBS foams.

## 1. Introduction

Lightweight elastomeric foams are increasingly attractive for applications requiring reduced mass together with deformation tolerance, energy absorption, and vibration damping. Their cellular architecture enables material usage to be reduced while retaining the mechanical functionality required under repeated deformation [1,2,3,4,5,6]. Thermoplastic elastomers are well suited to this design space because they combine melt processability with substantial reversible deformation [2,7]. Among them, styrene–butadiene–styrene (SBS) block copolymers offer a physically crosslinked morphology in which glassy styrenic domains act as physical junctions within a compliant polybutadiene-rich matrix, giving rise to a mechanical response that can be strongly modified through formulation-induced changes in matrix resistance [8,9,10,11]. This makes SBS an attractive platform for lightweight structures in which stiffness, extensibility, recovery, and damping must be balanced rather than maximized independently [7,9,10,11].

Thermally expandable microspheres provide a distinctive route to cellular SBS structures because each particle consists of a thermoplastic shell surrounding a volatile core that expands upon heating [12,13]. Expansion therefore occurs through the growth of discrete shell-containing particles rather than through unrestricted gas generation directly within the polymer matrix. Because the expanding gas remains confined within the thermoplastic shell, intact microspheres inherently generate discrete, closed cellular domains within the surrounding polymer matrix. This particle-confined expansion mechanism provides a route to density reduction while limiting direct gas transport through the matrix, although the preservation of the resulting cellular structure ultimately depends on the resistance of the surrounding polymer [7,12,13,14,15,16,17,18,19]. The surrounding polymer must deform sufficiently to accommodate shell growth while simultaneously providing enough resistance to retain the expanded geometry after processing.

In this context, processing oil should not be regarded solely as a conventional aid for plasticization and processability. By modifying the rheological resistance of the SBS matrix, additional oil can alter the mechanical constraint imposed on expanding microspheres and thereby influence the extent of expansion, the retention of the expanded geometry, and the resulting cellular architecture. Its role therefore extends beyond facilitating processing or promoting density reduction to controlling the balance between microsphere growth and structural stabilization. This balance suggests that microsphere foaming should not be described by a simple relationship in which decreasing matrix resistance continuously improves expansion. Excessive resistance can restrict microsphere growth and prevent the intended density reduction [7,14,18], whereas insufficient resistance may permit extensive deformation without providing adequate structural support for the expanded cellular architecture [7,18]. Stable foam formation is therefore expected to depend on the balance between matrix resistance, microsphere loading, and the imposed volumetric expansion demand [13,19]. However, the use of processing oil as a systematic rheological control parameter for governing this balance remains insufficiently established. This gap is particularly relevant for SBS, where oil-induced changes in the mobility of the rubbery phase and in the relative contribution of the styrenic physical domains can substantially alter the viscoelastic resistance encountered during particle growth [8,9,11].

A second limitation concerns the characterization of the resulting cellular state. Bulk density and two-dimensional pore-size measurements provide essential information, but neither alone can distinguish whether a change in pore size reflects an increase in total void fraction or redistribution of a similar void volume into larger or more heterogeneous cellular domains. Formulations with comparable density may therefore possess markedly different internal architectures [3,18,20,21]. Dielectric response offers a useful bulk-sensitive complement because the effective permittivity of a cellular polymer depends on the volumetric contribution of the low-permittivity gas phase as well as on the continuity and spatial distribution of the surrounding polymer phase [21,22,23,24,25,26]. Used together with microscopy and density measurements, dielectric characterization can therefore provide additional evidence regarding the volumetric character and continuity of the cellular structure. Mechanical evaluation likewise requires more than a single metric: load-bearing capacity, toughness, dimensional recovery, residual deformation, and hysteretic energy dissipation represent distinct aspects of performance and may favor different formulation states. A meaningful optimization strategy must therefore integrate structural, dielectric, quasi-static, and cyclic responses rather than relying on density reduction or pore size alone.

Accordingly, this study systematically varied processing-oil content, expandable-microsphere loading, and target density to determine how matrix rheological resistance governs microsphere expansion, cellular stabilization, and function-dependent performance in SBS foams. The experimental framework combined rheology, experimental density measurements, optical microscopy, dielectric characterization, tensile testing, and multi-day cyclic deformation analyses of thickness recovery, residual strain, and damping. The central hypothesis was that stable cellular development depends on the balance between matrix rheological resistance and the imposed expansion demand, and that greater pore expansion does not necessarily correspond to maximum mechanical performance. Particular attention was therefore given to separating morphology development from bulk void response and to distinguishing quasi-static strength, long-term dimensional retention, and cyclic energy dissipation as independent optimization criteria. The objective was to examine the relationships between matrix resistance, microsphere expansion, cellular structure, dielectric response, and function-specific mechanical behavior, rather than treating density reduction as the sole criterion of foam optimization. The novelty of this work lies in three aspects: (i) the additional processing oil in an already oil-extended SBS grade is used as a deliberate rheological control parameter for microsphere expansion rather than merely as a processing aid; (ii) optical microscopy is combined with dielectric permittivity as a volume-sensitive probe to separate morphology development from bulk void response; and (iii) quasi-static toughness, dimensional retention and cyclic damping are evaluated as independent, function-specific optimization criteria rather than treating density reduction as the sole objective.

## 2. Materials and Methods

### 2.1. Materials

Styrene–butadiene–styrene (SBS) triblock copolymer (Globalprene 1485, LCY Chemical Corp., Taipei City, Taiwan) was used as the polymer matrix. According to the supplier, the grade contains approximately 32 wt% styrene and 31 wt% processing oil, with a melt flow index of 8 g/10 min measured at 190 °C/5 kg and a Shore A hardness of 47. Thermally expandable microspheres (Expancel 920 DU 120, Nouryon, Göteborg, Sweden), with a reported median unexpanded particle size of 28–38 µm and an onset expansion temperature of 122–132 °C, were used as the foaming agent. A low-viscosity technical white mineral oil (Merlot 205, Petroyağ, Gebze, Türkiye) was additionally incorporated to systematically modify the rheological resistance of the SBS matrix. As the as-received grade is already oil-extended, the processing-oil content is distinguished in this study as inherent oil (31 wt% of the as-received SBS) and additional oil (10, 20 and 30 phr). The total oil content therefore corresponds to approximately 31.0, 37.3, 42.5 and 46.9 wt% for the as-received SBS, 10P, 20P and 30P, respectively.

### 2.2. Sample Preparation

Prior to melt processing, SBS pellets were dried in a vacuum oven at 60 °C for 6 h. In the first compounding stage, additional mineral processing oil was incorporated into SBS at 10, 20, and 30 phr, where phr denotes parts per hundred parts of as-received SBS (including its inherent oil). As-received SBS was processed under the same conditions and used as the unmodified reference for rheological characterization.

**Figure 1:**
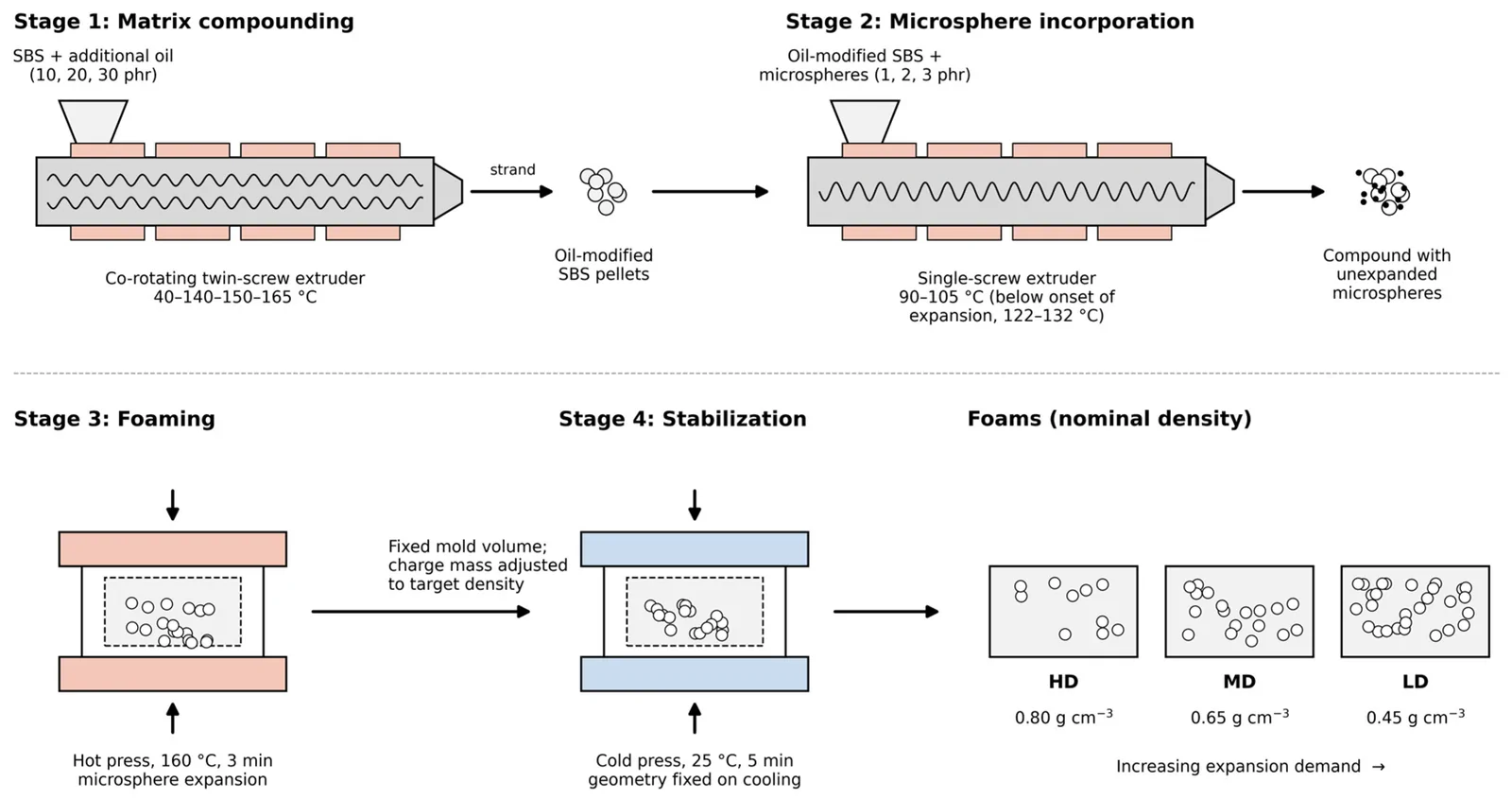
Schematic representation of the sample preparation: two-stage compounding (incorporation of additional processing oil into SBS, followed by incorporation of the expandable microspheres below their onset expansion temperature) and fixed-volume compression molding, in which the charge mass was adjusted to obtain the HD, MD and LD nominal target densities.

A two-stage compounding procedure was used to separate preparation of the oil-modified SBS matrix from the incorporation of the thermally expandable microspheres (Figure 1). In the first stage, SBS and additional processing oil were melt compounded using a co-rotating twin-screw extruder (RTX-T18, KÖKBİR, Lüleburgaz, Kırklareli, Türkiye) to obtain homogeneous oil-modified SBS compounds. The barrel-temperature profile was 40–140–150–165 °C. The extrudates were cooled and pelletized. In the second stage, thermally expandable microspheres were incorporated into these pre-compounded matrices at 1, 2, and 3 phr using a single-screw extruder (FX-20, Filamex, Istanbul, Türkiye). The extrusion temperature was maintained between 90 and 105 °C to minimize premature microsphere expansion during compounding, remaining below the reported onset expansion range of Expancel 920 DU 120 (122–132 °C). The resulting compounds were subsequently compression-molded at 160 °C to thermally activate the microspheres and generate the cellular structures used for further characterization.

Foaming and shaping were carried out by compression molding using a fixed mold cavity. The compounds were first hot-pressed at 160 °C for 3 min to activate the thermally expandable microspheres and generate the cellular structure. The mold was then transferred to a cooled press maintained at 25 °C and held for 5 min to stabilize the expanded geometry before demolding. The mass introduced into the mold was adjusted to impose nominal target densities of 0.80, 0.65, and 0.45 g cm^−3^, corresponding to the HD, MD, and LD conditions, respectively. Samples were coded as HD/MD/LD–x–y, where the first term denotes the nominal density condition, x represents the additional processing-oil content (10, 20, or 30 phr), and y represents the expandable-microsphere loading (1, 2, or 3 phr). For example, MD-10-3 denotes the formulation containing 10 phr additional processing oil and 3 phr expandable microspheres prepared at a nominal target density of 0.65 g cm^−3^. For concise discussion of formulation groups, B1, B2, and B3 are used to denote microsphere loadings of 1, 2, and 3 phr, respectively. Grouped expressions such as 10P-HD, 20P-MD, or 30P-LD refer to formulations containing the specified additional processing-oil content under the corresponding nominal density condition.

### 2.3. Rheological Characterization

The rheological response of the SBS matrices was characterized using a rotational rheometer (Discovery Hybrid Rheometer-3, DHR-3, TA Instruments, New Castle, DE, USA) equipped with 25 mm parallel plates. Measurements were performed at 160 °C using a constant gap of 1000 µm, corresponding to the compression-molding temperature employed during foam preparation. All rheological measurements were conducted on the unfoamed compounds obtained after the first compounding stage, prior to the incorporation of expandable microspheres, so that the intrinsic effect of processing-oil content on matrix rheology could be evaluated independently of microsphere expansion. Prior to frequency-sweep measurements, strain-sweep tests were performed to identify the linear viscoelastic region. Frequency sweeps were subsequently conducted from 600 to 0.1 rad s^−1^ at a constant strain amplitude of 1%, which remained within the linear viscoelastic region. The storage modulus (G′), loss modulus (G″), and loss factor (tan δ) were obtained as functions of angular frequency. Steady-shear measurements were additionally performed at 160 °C to determine the shear-rate-dependent viscosity of the SBS matrices and to quantify the change in matrix flow resistance with increasing processing-oil content. 

### 2.4. Experimental Density Measurements

The experimental densities of the foamed specimens were determined after conditioning for 24 h at room temperature to minimize post-molding dimensional changes. Measurements were performed using an analytical balance equipped with an Archimedes density-determination kit and isopropyl alcohol as the immersion medium. The liquid density was determined at the measurement temperature and used for calibration. For each formulation, specimens were cut from three different regions of the molded plaque, and the reported value represents the mean of three measurements. The nominal target density was defined as the ratio of the compound mass introduced into the mold to the fixed mold volume of 1.8 cm^3^, corresponding to 0.80, 0.65, and 0.45 g cm^−3^ for the HD, MD, and LD conditions, respectively.

### 2.5. Optical Microscopy and Pore-Size Analysis

The cellular morphology of the foamed specimens was examined using an optical microscope (SOIF BK5000). Cross-sectional images were acquired to evaluate pore development, size distribution, and structural integrity as a function of processing-oil content and nominal density level. Morphological comparisons were performed for the B3 formulations containing 3 phr expandable microspheres. Pore-size analysis was performed using ImageJ image-analysis software (version 1.54, National Institutes of Health, Bethesda, MD, USA). For each formulation, pore diameters were measured on one representative micrograph, corresponding to 45–105 pores per formulation, and the results are reported as mean pore size and coefficient of variation (CV).

### 2.6. Dielectric Characterization

The dielectric properties of the SBS-based formulations were characterized in the X-band using a vector network analyzer (VNA) (P9373A, Keysight Technologies, Santa Rosa, CA, USA) connected to a coaxial airline. The complex scattering parameters (S-parameters) were measured over the 8.2–12.4 GHz frequency range, and the complex relative permittivity was calculated using the Nicolson–Ross–Weir (NRW) method in its NRW-based polynomial-fit implementation, which suppresses the discontinuities of the conventional NRW solution that arise when the specimen thickness approaches integer multiples of half the wavelength in the sample. The real part of the relative permittivity (ε′) was used as the primary dielectric parameter for comparison among the formulations. For the density–permittivity analysis presented in the main text, ε′ at 10.03 GHz was selected as a representative value, while the complete frequency-dependent response was evaluated over the full X-band range. For each formulation, three independent measurements were performed, and the reported permittivity values represent their mean. Toroidal specimens with inner and outer diameters of 3 and 7 mm, respectively, were prepared using a custom-made cutting die, and the inner bore was finished with a dedicated tool so that each specimen slid onto the inner conductor and fitted snugly against the outer conductor without bending. The specimen thickness (i.e., its length along the airline axis) was 3 mm. Owing to this snug fit, no air-gap correction was applied. Specimens exhibiting tears, cracks, or other surface defects were excluded from the measurements. The system was calibrated with an electronic calibration kit before each measurement session.

### 2.7. Tensile Testing

Tensile tests were performed using an AHP universal testing machine equipped with tensile grips. Dumbbell-shaped specimens were cut from the molded plaques using an elastomer tensile-specimen cutting die. Measurements were conducted at room temperature at a constant crosshead speed of 10 mm min^−1^. For each formulation, at least four specimens were tested, and the results are reported as mean ± standard deviation. Toughness was calculated as the area under the engineering stress–strain curve from zero strain up to the fracture point by numerical integration. Only foamed specimens that retained sufficient geometric integrity after molding and expansion were considered suitable for tensile characterization.

### 2.8. Cyclic Deformation and Thickness-Recovery Tests

Cyclic compression tests were performed using an AHP universal testing machine equipped with a 500 N load cell. Only foamed formulations that reached their nominal target density and retained sufficient geometric integrity were included in the cyclic analysis; the LD series, which exhibited structural instability, was therefore excluded, restricting the analysis to stable HD and MD formulations. Each test specimen consisted of three circular discs (approximately 1.9–2.0 mm in thickness and 25 mm in diameter) cut from the molded plaques and stacked without adhesive, following the plied-specimen configuration described in ASTM D395 [27]. The same stacking configuration was used for all formulations. A preload of 0.1 N was applied, and force and displacement were zeroed at this contact point. The specimens were compressed by 15% of their initial height at a crosshead speed of 5 mm min^−1^ and subjected to 20 loading–unloading cycles per test day over three consecutive days, corresponding to 60 cycles in total. For each formulation, one specimen stack was tested, and the cycle-to-cycle evolution over the 60 cycles is reported. The Day 1 sequence was regarded as a conditioning stage for all formulations; Day 1 data were neither excluded nor corrected but are reported as recorded, while cross-formulation comparisons are based primarily on the conditioned Day 2–3 response. Between successive test days, the specimens were stored unloaded at room temperature for 24 h to allow recovery, and the stack height was measured before and after each daily test sequence to quantify thickness loss and delayed dimensional recovery. For each cycle, compressive stress was calculated as σ = F/A_0_, where F is the applied compressive force and A_0_ is the initial cross-sectional area, and strain as ε = d/h_0_, where d is the compressive displacement and h_0_ is the initial stack height. The maximum stress (σmax) was taken as the peak stress attained during each cycle. The dissipated energy was calculated from the area enclosed by the loading and unloading branches, Wdiss = Wload − Wunload, where Wload and Wunload are the areas under the respective stress–strain curves. The damping parameter was defined as the hysteretic loss ratio, η = Wdiss/Wload, and expressed as a percentage. Residual strain (εres) was defined as the strain remaining when the unloading stress returned to approximately zero (σ ≤ 0.001σmax). These parameters were used to characterize cyclic load-bearing capacity, hysteretic energy dissipation, and cycle-resolved residual deformation, respectively.

## 3. Results

### 3.1. Rheological Response of the SBS Matrix Prior to Foaming

**Figure 2:**
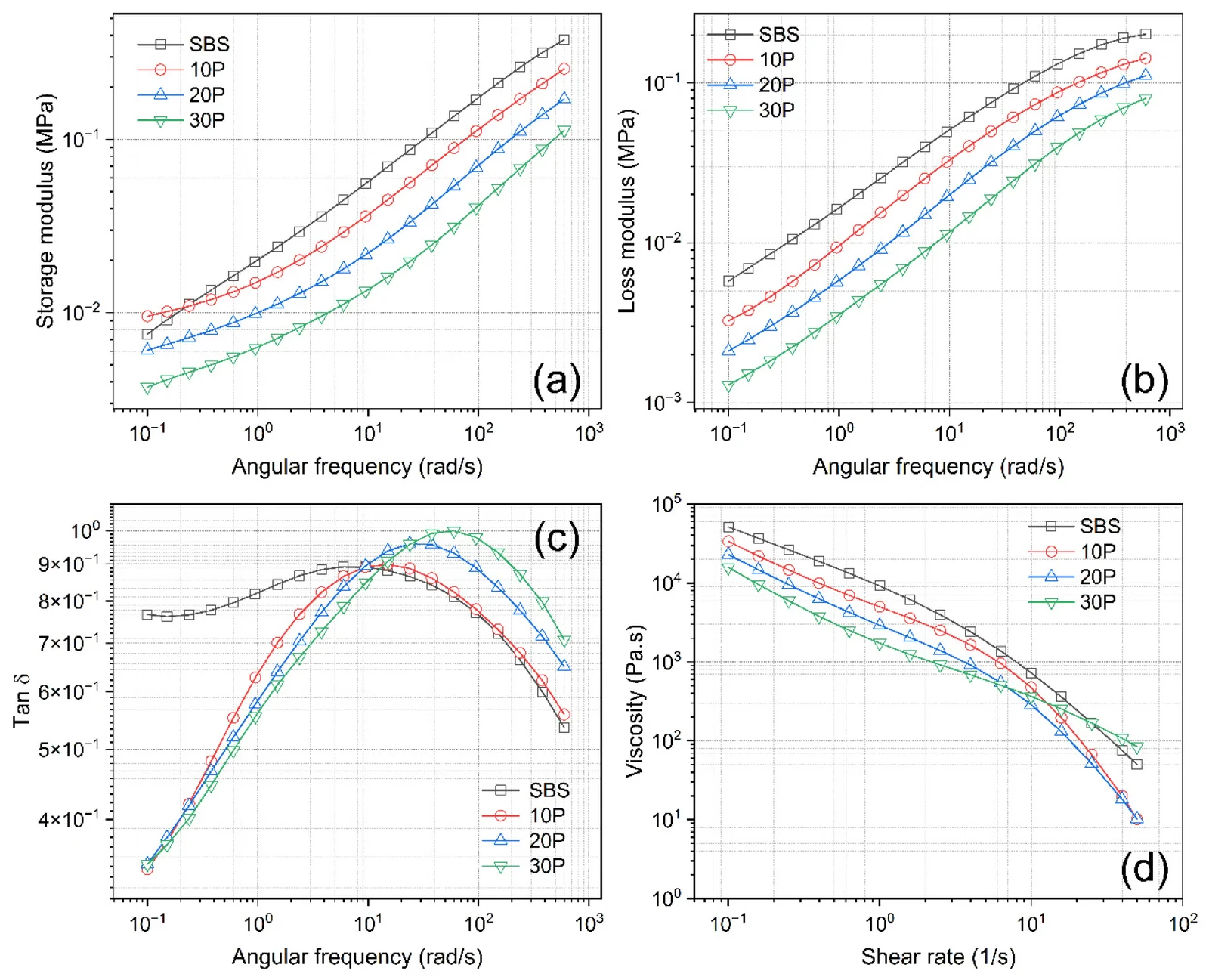
Effect of processing-oil content on the rheological behavior of SBS matrices prior to foaming: (**a**) storage modulus, G′, (**b**) loss modulus, G″, (**c**) tan δ, and (**d**) steady-shear viscosity.

Figure 2 shows the effect of processing-oil content on the rheological response of the SBS matrix prior to foaming. The combined evaluation of G′, G″, tan δ, and viscosity was used to establish the viscoelastic and flow resistance of the matrix as a function of oil content. Rather than serving only as an indication of plasticization, these measurements define the distinct rheological states of the 10P, 20P, and 30P formulations that are expected to influence the resistance encountered during subsequent expandable-microsphere growth, while as-received SBS provides the unmodified reference state.

As shown in Figure 2a, processing-oil addition altered not only the magnitude of G′ but also its frequency dependence. As-received SBS exhibited a pronounced decrease in G′ toward the low-frequency region, consistent with the increasing contribution of long-time relaxation processes. In contrast, the oil-containing formulations showed a progressively weaker frequency dependence at low frequencies and developed a more plateau-like response. This behavior may be associated with preferential plasticization of the PB-rich phase by the processing oil [8,28]. Since the as-received grade already contains 31 wt% oil, the PB-rich phase is already substantially plasticized, and the additional oil extends rather than initiates this plasticization. The term “preferential” refers here to the phase selectivity of mineral oil for the PB midblock reported in these studies, which is independent of the baseline oil level, whereas the incremental effect per unit of added oil is expected to decrease as the PB phase becomes increasingly diluted. By reducing the frictional and viscous contribution of the rubbery phase, oil addition can suppress the dominance of long-time matrix relaxation, while the comparatively less affected PS-rich physical domains continue to contribute to the elastic response. Accordingly, the low-frequency behavior should not be interpreted as the formation of a new network, but rather as an increased relative contribution of the pre-existing physical junctions within the experimentally accessible frequency window. At approximately 1 rad s^−1^, where G′ followed a clear monotonic ordering, the modulus decreased from about 17–18 kPa for SBS to 14–15 kPa for 10P, 10 kPa for 20P, and 6 kPa for 30P. This corresponds to an approximately threefold reduction between SBS and 30P and a 2.4-fold reduction from 10P to 30P, confirming that the oil-extended formulation range spans a substantial difference in elastic matrix resistance. Toward 0.1 rad s^−1^, however, this ordering was no longer preserved. G′ was approximately 7.5 kPa for SBS, 9.3 kPa for 10P, 6.1 kPa for 20P, and 3.7 kPa for 30P. The crossover between SBS and 10P at the lowest frequencies indicates that processing oil does not simply produce a uniform downward shift in modulus, but also modifies the balance between long-time matrix relaxation and the contribution of the SBS physical-domain structure.

The corresponding loss-modulus response in Figure 2b showed a more systematic dependence on processing-oil content, with G″ decreasing over the entire frequency range in the order SBS > 10P > 20P > 30P. Unlike G′, no comparable low-frequency redistribution or plateau-like tendency was observed. This absence is consistent with the predominantly elastic nature of the SBS physical-domain contribution inferred above, whose low-frequency signature is expected to appear primarily in G′ rather than in the dissipative response. Since both G′ and G″ decreased with oil addition, the overall effect should therefore be interpreted as a reduction in the viscoelastic resistance of the SBS matrix rather than as a simple shift toward a more elastic response.

The corresponding tan δ behavior in Figure 2c further reveals that processing oil modifies the relative balance between the elastic and dissipative components of the SBS matrix in a strongly frequency-dependent manner. At low frequencies, SBS exhibited a substantially higher tan δ value, approximately 0.76 at 0.1 rad s^−1^, whereas the oil-containing formulations converged to a much lower range of about 0.33–0.34. This behavior is consistent with the increased relative contribution of the pre-existing physical-domain structure inferred from the low-frequency G′ response. As the frequency increased, tan δ increased markedly for all oil-containing formulations, and the position of the maximum shifted progressively toward higher frequencies with increasing oil content, from approximately 15–20 rad s^−1^ for 10P to about 30–40 rad s^−1^ for 20P and 50–70 rad s^−1^ for 30P. Taking the characteristic relaxation time as τ ≈ 1/ω_max, this shift corresponds to a reduction in τ from approximately 0.05–0.07 s for 10P to 0.025–0.033 s for 20P and 0.014–0.020 s for 30P, i.e., an approximately three- to fourfold acceleration of the dominant relaxation process, consistent in direction and magnitude with the reductions in G′ and viscosity. The maximum tan δ value also increased slightly from approximately 0.89 for 10P to about 0.95 for 20P and nearly 0.99 for 30P. This frequency-dependent redistribution may be associated with the preferential plasticization of the PB-rich phase, which modifies the relative contributions of the rubbery matrix and the less affected styrenic physical domains to the overall viscoelastic response. Accordingly, processing oil does not produce a uniform shift toward either a more elastic or a more viscous state but modifies the balance between these contributions across the investigated frequency range.

The steady-shear viscosity response in Figure 2d confirms the progressive reduction in matrix flow resistance with increasing processing-oil content. At low shear rates, the viscosity followed the order SBS > 10P > 20P > 30P, with approximate values at 0.1 s^−1^ of 5.0 × 10^4^, 3.3 × 10^4^, 2.4 × 10^4^, and 1.6 × 10^4^ Pa·s, respectively. This corresponds to an approximately threefold reduction between SBS and 30P and about a twofold reduction across the oil-extended formulation range from 10P to 30P. All formulations exhibited pronounced shear-thinning behavior, and the differences between the curves progressively diminished with increasing shear rate. At higher shear rates, the curves approached one another and partially crossed, indicating that the influence of processing oil on viscosity is strongly shear-rate-dependent rather than representing a uniform downward shift over the entire flow regime. The combined decrease in G′ and viscosity confirms that increasing processing-oil content reduces both the elastic and flow resistance of the SBS matrix, which is expected to influence the subsequent microsphere-expansion behavior.

### 3.2. Density-Controlled Expansion Response

**Figure 3:**
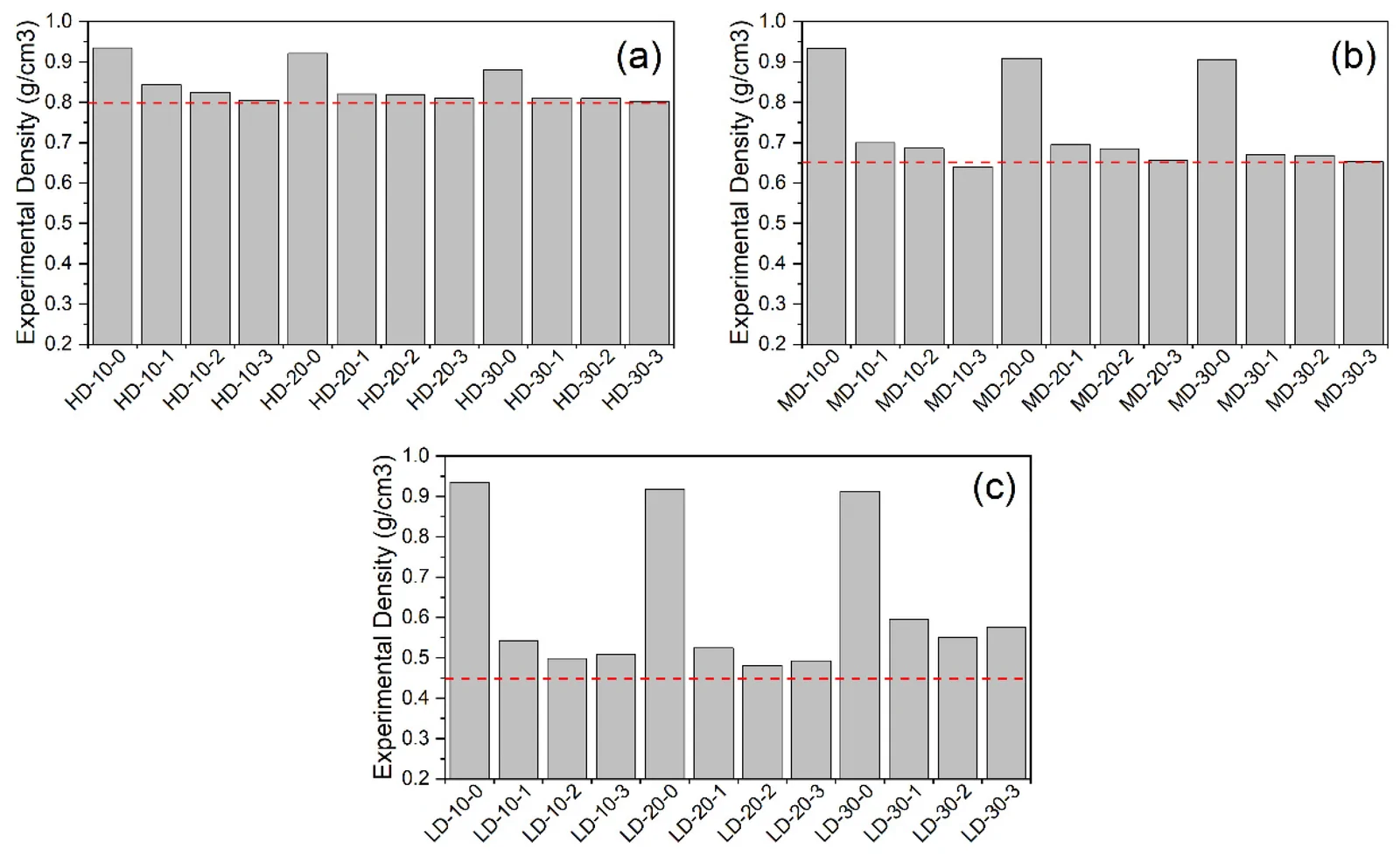
Experimental densities of SBS-based formulations prepared at different nominal target density levels: (**a**) HD, 0.80 g cm^−3^, (**b**) MD, 0.65 g cm^−3^, and (**c**) LD, 0.45 g cm^−3^.

Figure 3 compares the experimentally measured densities of the SBS-based formulations with the nominal target densities defined by the fixed-volume molding design. The red dashed lines indicate the target densities of 0.80, 0.65, and 0.45 g cm^−3^ for the HD, MD, and LD series, respectively. Because the mold volume was kept constant, decreasing the nominal target density increased the volumetric expansion required from the formulation. The three density levels therefore represent progressively more demanding expansion conditions, allowing the combined influence of processing-oil content and expandable-microsphere loading on macroscopic density attainment to be evaluated. Under fixed-volume molding, the measured density reflects the expanded volume retained after cooling: the microsphere loading determines the total gas volume available for expansion, whereas the matrix resistance governs how far each microsphere can expand against the surrounding SBS and whether this expansion is retained after cooling.

Before considering the contribution of microsphere expansion, the density variation in the microsphere-free reference should be noted. In the absence of expandable microspheres, the measured density decreased progressively with increasing processing-oil content, from approximately 0.934 g cm^−3^ for 10P to about 0.92 g cm^−3^ for 20P and 0.881 g cm^−3^ for 30P. This decrease reflects the increasing fraction of the lower-density processing oil in the SBS-based compound and therefore represents a formulation-induced density reduction independent of foaming.

For the HD series (Figure 3a), the measured density progressively approached the nominal target of 0.80 g cm^−3^ as the expandable-microsphere loading increased. At each oil content, the addition of microspheres reduced the measured density relative to the corresponding microsphere-free reference, indicating that a larger microsphere loading generated a greater macroscopic expansion response. This effect was most evident for the more resistant 10P matrix, where 1 and 2 phr microspheres reduced the density only to approximately 0.843 and 0.825 g cm^−3^, respectively, whereas 3 phr was sufficient to reach the target level. Increasing the processing-oil content provided an alternative route to the same outcome by lowering the matrix resistance; for example, at 1 phr microsphere loading, the density decreased from approximately 0.843 g cm^−3^ for 10P to about 0.812 g cm^−3^ for 30P. At 3 phr, all 10P, 20P, and 30P formulations converged close to 0.80 g cm^−3^, indicating that under the relatively modest HD expansion requirement, the highest microsphere loading investigated could largely compensate for differences in matrix resistance. For the MD series (Figure 3b), lowering the nominal target density to 0.65 g cm^−3^ increased the required degree of expansion and made the balance between matrix resistance and microsphere loading more critical. The 10P formulation remained above the target at 1 and 2 phr microsphere loading, with measured densities of approximately 0.700 and 0.686 g cm^−3^, respectively, whereas 3 phr reduced the density to the target range. A similar trend was observed for the 20P series, although the lower matrix resistance enabled a closer approach to the nominal density at the same microsphere loadings. The effect of matrix plasticization was most evident for 30P, where even 1 and 2 phr microspheres reduced the density to approximately 0.670 and 0.668 g cm^−3^, respectively, before the B3 formulation converged close to the 0.65 g cm^−3^ target.

The LD series (Figure 3c) exhibited a fundamentally different response. None of the formulations reached the nominal target of 0.45 g cm^−3^, and the monotonic decrease in density with increasing microsphere loading observed under HD and MD conditions was no longer preserved. In both the 10P and 20P series, the minimum density was obtained at 2 phr, while increasing the microsphere content to 3 phr produced no additional density reduction and, in some cases, resulted in a slight increase. More strikingly, the 30P formulations showed consistently higher densities than the corresponding 10P and 20P foams, despite having the lowest matrix resistance. Thus, under the highest expansion demand, further matrix softening no longer promoted greater retained expansion; instead, the 30P-LD series provided the first macroscopic indication that the matrix had become insufficiently resistant to stabilize the expanded structure. The non-monotonic B2–B3 response can be related to the onset of over-expansion-induced structural instability. Once the expansion demand exceeds the stabilizing capability of the surrounding SBS matrix, additional microsphere expansion may promote shell or cell rupture, coalescence-like deformation, or loss of retained cellular volume rather than a further stable increase in specimen volume. In this regime, increasing either microsphere loading or processing-oil content no longer provides the benefit observed under HD and MD conditions. The LD results therefore indicate the onset of a less stable expansion regime, with the 30P-LD formulations showing the clearest signs of reduced cellular retention. Within the investigated range, increasing microsphere loading was therefore the more effective route to the target density under HD and MD conditions while preserving or even improving strength, whereas additional oil mainly provided a secondary density reduction at the expense of matrix stiffness, and neither route was sufficient under LD conditions. The structural origin of this behavior is examined more directly through cellular morphology in the following section.

### 3.3. Cellular Morphology and Pore Development

**Figure 4:**
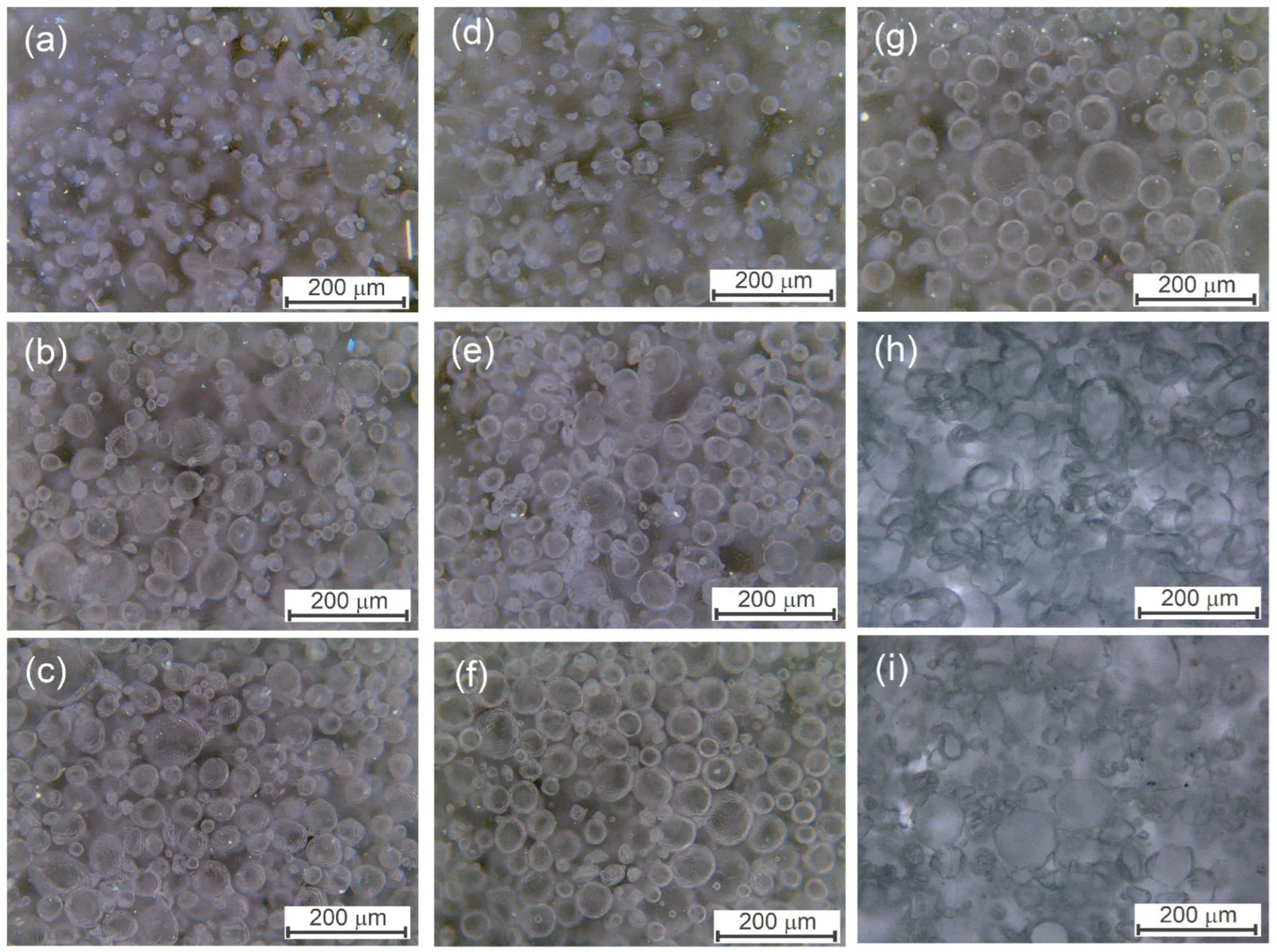
Optical micrographs of SBS foams containing 3 phr expandable microspheres as a function of processing-oil content and nominal density level: (**a**) HD-10-3, (**b**) MD-10-3, (**c**) LD-10-3, (**d**) HD-20-3, (**e**) MD-20-3, (**f**) LD-20-3, (**g**) HD-30-3, (**h**) MD-30-3, and (**i**) LD-30-3. The columns correspond to 10P, 20P, and 30P, while the rows represent the HD, MD, and LD conditions, respectively.

Figure 4 presents the optical micrographs of the SBS foams containing 3 phr expandable microspheres, arranged to enable direct comparison of both nominal density level and processing-oil content. The rows correspond to the HD, MD, and LD conditions, while the columns represent 10P, 20P, and 30P, respectively. This arrangement allows the cellular morphology to be evaluated under a constant microsphere loading while independently tracking the effects of increasing expansion demand and decreasing matrix resistance. Across the three density levels, the cellular morphology evolved from relatively small and well-defined pores under HD conditions to larger and more extensively expanded pores under MD conditions, while the LD series showed increasing heterogeneity, particularly at higher processing-oil contents. Within the HD series, all B3 formulations retained clearly distinguishable cellular structures, but the local pore development changed markedly with processing-oil content. The average pore size increased from 22.82 µm for HD-10-3 to 36.19 and 42.36 µm for HD-20-3 and HD-30-3, respectively. Despite their similar bulk densities, the HD formulations exhibited markedly different pore sizes, indicating that density alone does not fully describe the cellular morphology. The trend is consistent with the rheological gradient established in Figure 2, where increasing processing-oil content progressively reduced the elastic and flow resistance of the SBS matrix. Under the relatively modest HD expansion requirement, this reduction in matrix resistance therefore facilitated greater local microsphere expansion without substantially altering the final bulk density. Importantly, however, two-dimensional optical microscopy cannot determine whether the larger pores observed at nearly constant density correspond to a true increase in bulk void fraction or primarily to a redistribution of a similar void volume into larger cellular domains. This distinction requires a volume-sensitive response and is examined further through the dielectric measurements.

The effect became more pronounced under the MD condition. The average pore size increased from 25.23 µm for MD-10-3 to 42.72 µm for MD-20-3 and 49.56 µm for MD-30-3. At the same time, the coefficient of variation (CV) decreased from 38.60% to 31.95% and 27.72%, respectively. Because the rheological measurements were performed at the foaming temperature of 160 °C, this trend can be related directly to the matrix resistance during expansion: the increase in mean pore size from 25.2 to 49.6 µm across the MD series coincided with a decrease in G′ at 1 rad s^−1^ from approximately 14–15 to 6 kPa and in the low-shear viscosity from 3.3 × 10^4^ to 1.6 × 10^4^ Pa·s. The simultaneous increase in pore size and reduction in relative size dispersion indicate that the enhanced pore development did not arise from random or uncontrolled enlargement. Instead, the reduction in matrix resistance allowed more extensive microsphere growth while the surrounding SBS phase remained sufficiently resistant to preserve a comparatively uniform cellular structure. Although the MD-30-3 micrograph exhibits lower optical contrast due to the presence of larger and thinner-walled cellular regions, the quantitative analysis showed that this formulation exhibited the largest mean pore size together with the lowest CV within the MD series. Among the examined formulations, MD-30-3 therefore combined the largest mean pore size with the lowest CV within the MD series; however, these morphological characteristics did not translate into the highest mechanical performance.

The LD condition revealed the limit of this progression. The LD-10-3 and LD-20-3 samples still retained clearly identifiable cellular boundaries, with average pore sizes of 47.5 and 47.4 µm and CV values of 38.2% and 31.2%, respectively, i.e., size dispersions comparable to those of the corresponding MD formulations. For LD-30-3, in contrast, the average pore size decreased to 38.29 µm and the CV increased sharply to 49.74%, despite the greater nominal expansion demand. This contrast with MD-30-3 is particularly informative because the matrix composition and microsphere loading were identical and only the imposed expansion demand differed. The reduction in pore size together with the pronounced increase in pore-size dispersion indicates that the additional expansion required under LD conditions was not retained as further controlled pore growth. Instead, the combined effect of high expansion demand and low matrix resistance appears to have exceeded the stabilizing capability of the SBS phase, promoting partial collapse, coalescence-like deformation, or loss of pore-boundary integrity. This interpretation is consistent with the density response in Figure 3, where the LD-30 formulations, despite having the lowest matrix resistance, retained less macroscopic expansion than the corresponding LD-10 and LD-20 formulations.

### 3.4. Density–Permittivity Relationship

**Figure 5:**
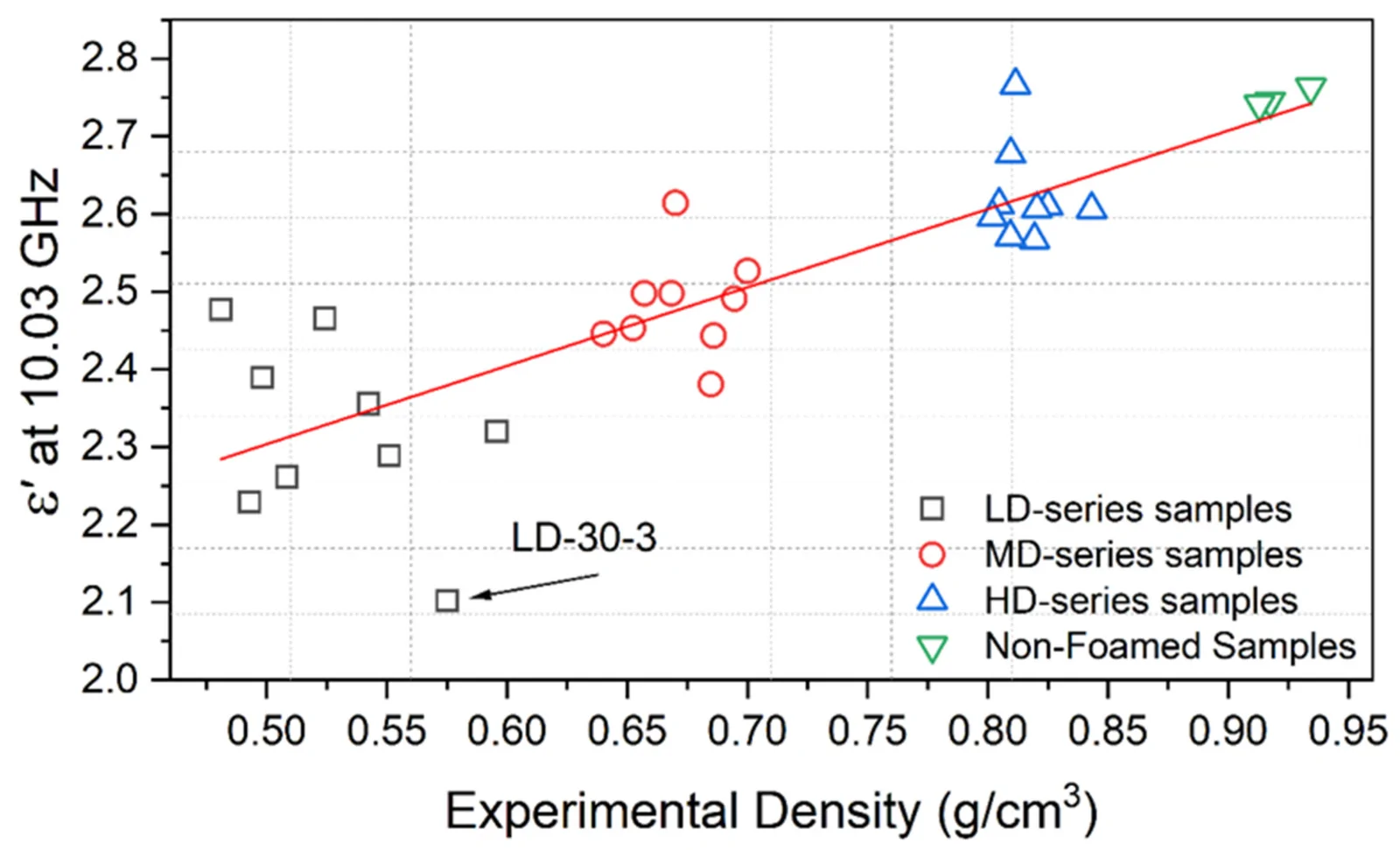
Experimental density dependence of the relative permittivity (ε′) of the SBS-based formulations measured at 10.03 GHz. Symbols represent the non-foamed, HD, MD, and LD formulations, and the solid line indicates the global empirical linear trend across the complete dataset.

Dielectric measurements were used as a bulk-sensitive complement to optical microscopy to determine whether differences in local pore morphology, particularly among formulations with comparable density, were accompanied by corresponding changes in the effective volumetric void response. Although the relative permittivity, ε′, was measured over the full 8.2–12.4 GHz frequency range, the comparison in Figure 5 is presented at 10.03 GHz to provide a common representative frequency for all formulations. This approach enables direct comparison among the formulations while avoiding the visual complexity of multiple overlapping spectra. As shown in Figure 5, ε′ generally increased with experimental density across the complete dataset. The non-foamed samples occupied the highest-density and highest-permittivity region, whereas the HD, MD, and LD formulations progressively shifted toward lower density and lower ε′ values. Despite differences in processing-oil content, microsphere loading, and nominal density level, the complete dataset therefore exhibited a consistent positive density–permittivity trend. An additional feature of this relationship is the progressive narrowing of the ε′ distribution with increasing density. While the low-density formulations exhibited a broader spread in ε′, the values converged within a comparatively narrower range at higher densities. This behavior may reflect the decreasing volumetric contribution of the cellular gas phase as the polymer fraction increases, thereby reducing the sensitivity of the effective dielectric response to differences in cellular structure among formulations. The linear fit shown in Figure 5 is included only as a global empirical guide to this overall trend across all measured samples and is not intended to represent a specific dielectric mixing model. The progressive reduction in ε′ with decreasing density is consistent with the increasing contribution of low-permittivity air to the effective bulk response of the cellular material. More importantly, the smooth density-dependent evolution of ε′ across the stable formulations, without an abrupt discontinuity or threshold-like change, is consistent with a predominantly discrete and closed-cell morphology rather than the development of a continuous interconnected void structure. This interpretation is also compatible with the expandable-microsphere-based foaming mechanism, in which individual expanded particles are expected to remain largely isolated within the surrounding SBS matrix.

The comparison with the optical-microscopy results further shows that average pore size and total effective void fraction should not be treated as equivalent structural parameters. This distinction is particularly evident in the HD series. As discussed in Figure 4, the average pore size increased from approximately 22.8 µm for HD-10-3 to 42.4 µm for HD-30-3, while the corresponding formulations remained within a relatively narrow macroscopic density range. Despite this pronounced difference in local pore dimensions, their ε′ values remained broadly consistent with the same density-dependent trend. The increase in average pore size therefore does not indicate a proportional increase in total bulk porosity. Instead, the reduced matrix resistance associated with increasing processing-oil content appears to redistribute the available void volume into larger cellular domains while leaving the overall volumetric air fraction comparatively similar. In this respect, ε′ provides a bulk-sensitive complement to the two-dimensional optical observations. The MD formulations showed the same overall behavior, with larger and relatively more uniform pores developing while the dielectric response remained broadly governed by the experimental density.

The LD formulations occupied the lowest-density region of the dataset and generally continued the decrease in ε′ observed from the non-foamed, HD, and MD samples. One formulation, however, exhibited a markedly different response. LD-30-3 showed an ε′ value of approximately 2.10 at an experimental density of about 0.575 g cm^−3^, placing it well below the global density–permittivity trend. Importantly, this unusually low value cannot be interpreted as evidence of a greater uniformly distributed bulk porosity, because other LD samples with comparable or even lower experimental densities exhibited substantially higher ε′ values. The deviation of LD-30-3 is instead consistent with the structural instability identified independently from the density and optical-microscopy results. The corresponding LD-30-3 formulation showed limited retained expansion, reduced pore-boundary definition, and increased pore-size heterogeneity. These observations indicate that the low ε′ value is more plausibly associated with the dimensional and surface irregularity generated by cellular instability than with an increase in stable internal void fraction. Because all specimens were cut with the same die and fitted snugly in the airline, a preparation-related interfacial gap is unlikely to account for this deviation, and the low value was reproduced in all three independent measurements. The additional air is therefore attributed to the material itself: the coarse and unstable cellular structure of LD-30-3 leaves larger cut-open pores at the specimen surfaces in contact with the conductors, thereby lowering the apparent ε′. Accordingly, the low ε′ of LD-30-3 is regarded as consistent with, rather than as independent proof of, the structural instability established from the density and optical-microscopy results.

Overall, the dielectric response indicates that ε′ primarily follows the bulk density of the SBS foams, while deviations from this trend are associated with structurally unstable formulations. The smooth density-dependent behavior of the stable formulations is consistent with a predominantly closed and discrete cellular architecture, whereas the anomalous response of LD-30-3 is consistent with the view that excessive expansion demand compromises stable cellular retention.

### 3.5. Tensile Response and Mechanical Stability of the Cellular Structure

**Figure 6:**
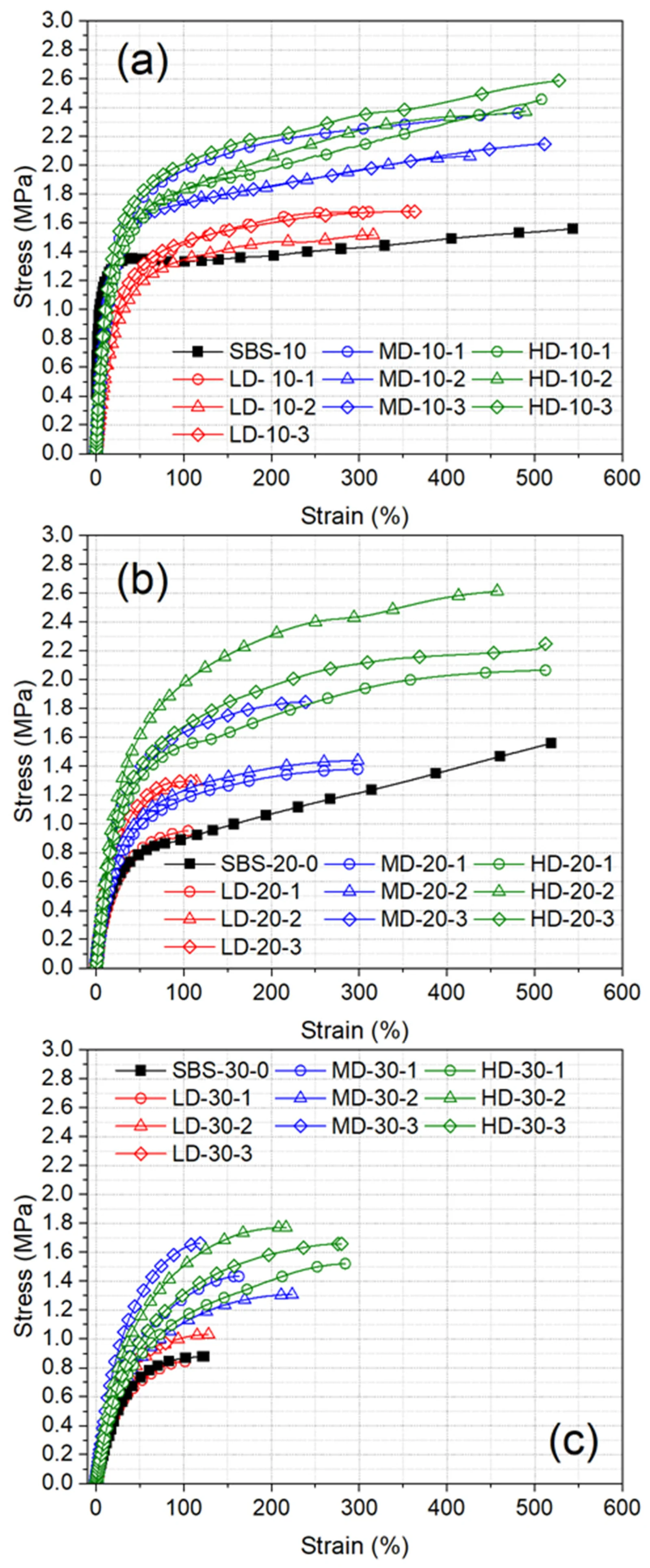
Stress–strain curves of SBS-based formulations with different processing-oil contents and nominal density levels: (**a**) 10P, (**b**) 20P, and (**c**) 30P.

The tensile behavior of the SBS-based formulations was evaluated both in terms of conventional mechanical performance and as an indirect indicator of the solid-state integrity of the cellular architecture. Although tensile loading does not isolate cellular stability from other contributions such as matrix extensibility, local ligament defects, and interfacial deformation, it provides a sensitive measure of how effectively the expanded structure maintains load-bearing continuity under large deformation. Figure 6 compares the stress–strain responses of the 10P, 20P, and 30P formulations in panels (a), (b), and (c), respectively, with the corresponding non-foamed matrix included as a reference in each panel.

Across the three oil levels, the overall stress response decreased progressively from 10P to 20P and 30P, in agreement with the reduction in matrix resistance established in Figure 2. The stress–strain curves therefore confirm that the softening induced by processing oil is retained in the solid-state response. At the same time, the foamed formulations frequently sustained stresses comparable to or higher than those of the corresponding non-foamed references. This behavior cannot be attributed to void formation itself, since increasing the void fraction would generally be expected to reduce the effective load-bearing area. Instead, it suggests that the expanded-microsphere phase modifies the load-transfer mechanism within the SBS matrix. A reinforcement-like contribution from the retained microsphere shells, together with strain hardening and redistribution of deformation through the surrounding polymer ligaments, may increase the apparent stress response. These contributions, however, compete with the opposing effects of ligament thinning and local stress concentration as the extent of cellular expansion increases. Since the individual contributions of shell reinforcement and matrix deformation cannot be separated from the present tensile data, the observed response is most appropriately interpreted as the net result of these competing mechanisms rather than as evidence of a single reinforcement mechanism.

The influence of nominal density level is apparent within each oil series. The HD formulations generally sustained the highest stresses and the largest deformation ranges, whereas the MD and particularly the LD samples showed progressively lower stress capacity and earlier termination. For the 10P and 20P systems, foaming did not produce a substantial increase in the maximum attainable strain relative to the non-foamed references; instead, the principal effect was the ability of several foamed formulations to sustain higher stresses over a comparable strain range. A different response was observed for 30P, where the non-foamed matrix failed at a comparatively low strain, while several HD and MD foams extended the accessible deformation range despite the lower absolute stress level. This distinction indicates that the mechanical consequence of cellular expansion depends strongly on the initial matrix state: in the more resistant 10P and 20P matrices, the primary benefit is expressed through load-bearing efficiency, whereas in the highly plasticized 30P matrix, the cellular architecture can additionally modify the deformation path and delay macroscopic failure under suitable expansion conditions. The dependence on microsphere loading was likewise not universally monotonic, providing further evidence for the competition between reinforcement-like contributions and structural weakening. Under the relatively resistant and moderately expanded 10P-HD condition, increasing microsphere content generally promoted the stress response, consistent with a regime in which the added cellular phase can contribute to load transfer without critically compromising the surrounding polymer ligaments. At higher oil contents and/or greater expansion demands, however, this trend became less systematic. In the 30P formulations, for example, the highest microsphere loading did not consistently produce the highest stress response, indicating that the benefit associated with the expanded-microsphere phase was increasingly offset by reduced ligament thickness, greater local deformation, and stress concentration. The non-monotonic B1–B3 response should therefore be viewed as a characteristic of the balance between these opposing effects rather than as an inconsistency in the mechanical behavior.

The comparison between MD-30-3 and the corresponding LD condition is particularly informative. MD-30-3 exhibited extensive pore expansion together with relatively low pore-size dispersion in Figure 4, yet these morphological characteristics did not translate into the highest tensile response. The same reduction in matrix resistance that facilitated extensive and comparatively uniform pore development also reduced the load-bearing capacity of the polymer framework. Thus, extensive pore expansion does not necessarily correspond to the mechanically optimal state. At the same time, the 30P-HD and 30P-MD formulations should be distinguished from the 30P-LD regime. The former remain mechanically functional despite their lower stress level and retain substantial deformability, whereas the latter combine a highly softened matrix with the most demanding expansion condition and exhibit a much earlier loss of mechanical continuity. This deterioration under LD conditions is consistent with the structural instability identified independently in the preceding analyses. In particular, the 30P-LD formulations combine limited retained expansion in Figure 3, disrupted and heterogeneous cellular morphology in Figure 4, anomalous dielectric behavior in Figure 5, and premature termination of the stress–strain response in Figure 6. These observations converge on the same interpretation: once the expansion demand exceeds the stabilizing capability of the highly plasticized matrix, irregular or partially disrupted cellular regions increasingly act as local stress concentrators and shorten the continuous load-transfer paths through the material. The stress–strain curves therefore illustrate the mechanical consequences of the rheology–expansion balance identified earlier. The quantitative effects of these competing mechanisms on tensile strength, strain at break, modulus, and toughness are examined in the following analysis.

The quantitative tensile parameters summarized in Table 1 confirm that the mechanical response is governed by a competition between the load-bearing contribution of the cellular/microsphere structure and the weakening associated with increasing expansion. For the non-foamed references, increasing processing-oil content reduced the elastic modulus sharply from 23.23 MPa for SBS-10 to 5.66 and 3.94 MPa for SBS-20 and SBS-30, respectively, whereas the corresponding maximum stress decreased more moderately from 1.761 to 1.744 and 1.053 MPa. This confirms that processing oil affected stiffness more strongly than strength and identifies the 10P matrix as a distinctly more resistant solid-state structure. For the foamed formulations, however, the measured modulus reflects not only matrix stiffness but also void fraction, cellular geometry, microsphere-shell contribution, and the resulting load-transfer path.

A particularly clear density-dependent crossover was observed in maximum stress. At all three oil levels, the HD foams exceeded the corresponding non-foamed reference, whereas the LD formulations remained below or approximately equal to it. For example, σmax increased from 1.761 MPa for SBS-10 to 2.283–2.531 MPa for the 10P-HD series, while remaining at 1.504–1.703 MPa under LD conditions. The same crossover was observed for the 20P and 30P systems. This systematic behavior is consistent with the proposed competing-mechanisms framework: under moderate expansion, the load-bearing contribution of the expanded microsphere/cellular structure outweighs the loss in effective ligament area, whereas under severe expansion the weakening associated with thinner and less stable load-bearing regions becomes dominant.

Toughness further separates stiffness and strength from total mechanical energy absorption. The 20P-HD formulations, for example, retained relatively low moduli of approximately 5.8–6.4 MPa while exhibiting toughness values of 8.72–9.65 MJ m^−3^ because of their high strain capacity. Relative to the non-foamed SBS-20 reference, foaming under HD conditions thus increased toughness from 5.91 MJ m^−3^ to 8.72–9.65 MJ m^−3^. The highest mean toughness was obtained for HD-10-3 at 11.50 MJ m^−3^, together with σmax = 2.531 MPa and a strain at break of 520%. Conversely, the 30P-HD formulations demonstrate that a highly softened matrix can still be mechanically functional when the cellular structure remains stable: foaming increased the toughness of SBS-30 from 0.96 MJ m^−3^ to approximately 3.6–3.9 MJ m^−3^ and extended the strain at break from 118% to 259–298%.

At higher expansion demand, the balance shifted progressively toward structural weakening. This transition was already evident in the 20P-LD series, where the strain at break decreased to 119–85% and toughness remained near 0.8–1.2 MJ m^−3^. The 30P formulations showed the clearest non-monotonic response. Under MD conditions, the 2 phr formulation exhibited a higher toughness than the 3 phr formulation (2.18 and 1.40 MJ m^−3^, respectively), even though MD-30-3 exhibited the largest mean pore size together with the lowest CV within the MD series. Under LD conditions, toughness changed from 0.75 to 1.10 and then to 0.54 MJ m^−3^ from B1 to B3. This B2–B3 reversal is consistent with the loss of stable expansion identified from the density and morphology results and indicates that increasing microsphere loading beyond an intermediate level does not necessarily improve mechanical performance.

The most severe response was therefore observed for LD-30-3, with a strain at break of 79% and a toughness of only 0.54 MJ m^−3^. Together with its limited retained expansion, disrupted cellular morphology, and anomalous dielectric response, this mechanical deterioration identifies the 30P-LD regime as the clearest manifestation of expansion-induced instability, although the onset of this behavior is already visible in the 20P-LD series. Overall, the tensile results demonstrate that the formulation promoting the most extensive pore development is not necessarily the one maximizing quasi-static mechanical performance. The highly plasticized 30P formulations remain relevant because their lower matrix resistance, while unfavorable for strength under severe expansion, may provide advantages in deformation recovery and damping, which are evaluated in the subsequent cyclic tests.

### 3.6. Cyclic Deformation, Recovery, and Energy Dissipation

**Figure 7:**
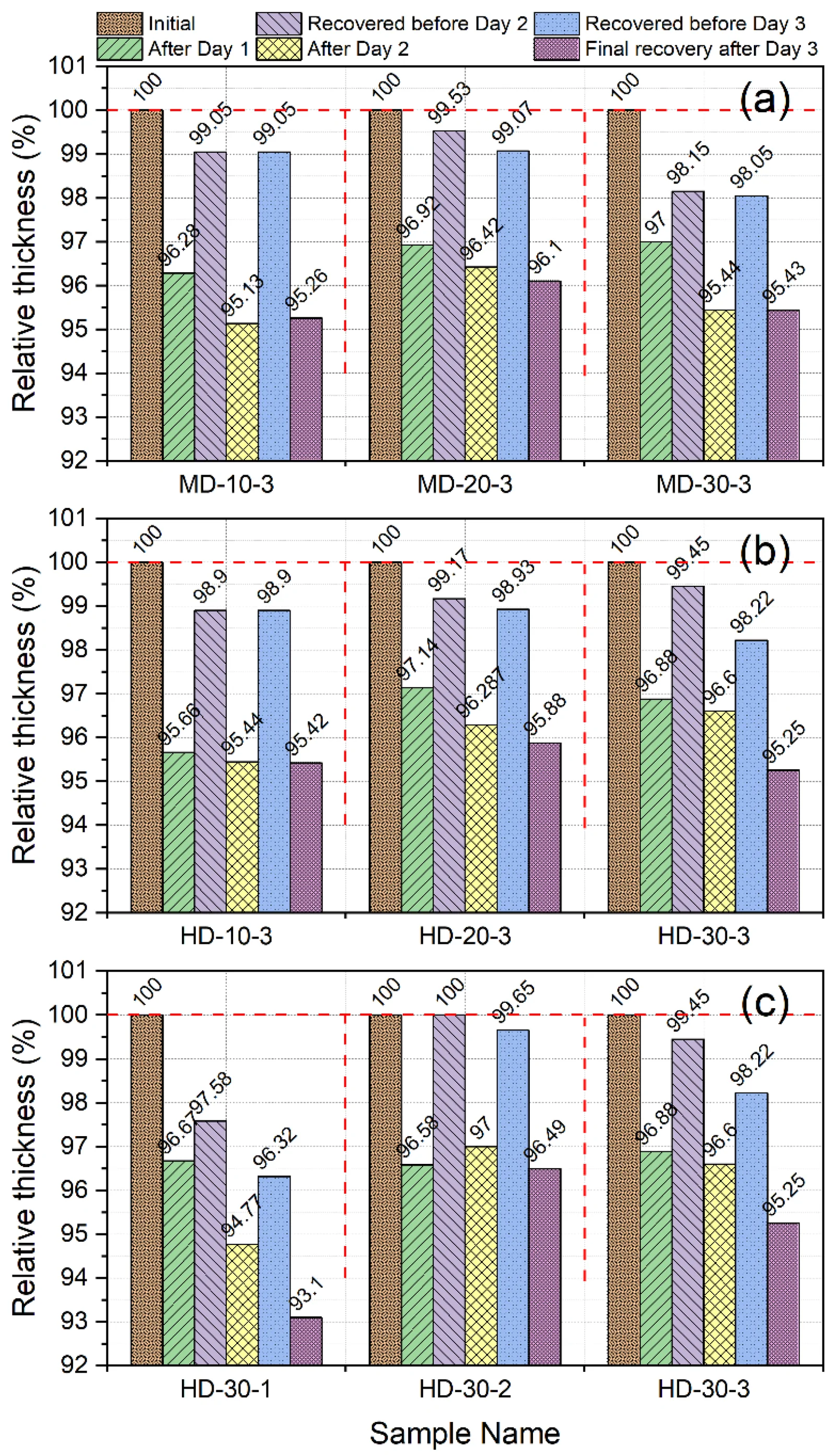
Relative thickness evolution of selected SBS foams during cyclic deformation and recovery over three consecutive test days: (**a**) B3-MD series, (**b**) B3-HD series, and (**c**) 30P-HD series. Initial specimen thickness was normalized to 100%.

The cyclic response was first evaluated through the evolution of specimen thickness during repeated deformation and subsequent recovery periods. Cyclic tests were performed only on formulations that achieved their nominal target density, thereby restricting the analysis to cellular structures that successfully reached the intended macroscopic expansion level. In Figure 7, the initial thickness of each specimen was normalized to 100%; values measured immediately after each loading day represent thickness retention after cyclic deformation, whereas the measurements obtained before the following test day quantify delayed recovery during the unloaded resting period. The final value after Day 3 therefore reflects the accumulated dimensional retention after the complete loading sequence. Panel (a) compares the effect of processing-oil content for the B3-MD formulations, panel (b) presents the corresponding B3-HD formulations, and panel (c) isolates the effect of microsphere loading within the 30P-HD series.

For the B3 formulations, both the MD and HD series showed substantial delayed recovery after each loading period, confirming that the immediate thickness loss produced during cyclic deformation was not entirely irreversible. In the MD series, the final retained thickness was highest for MD-20-3 at approximately 96.1%, whereas MD-30-3 did not provide the best long-term retention. A similar distinction was observed under HD conditions. HD-30-3 exhibited the highest recovery before Day 2, reaching approximately 99.45% of its initial thickness, but its final retained thickness after Day 3 decreased to approximately 95.25%, the lowest value among the three HD-B3 formulations. Thus, increasing processing-oil content can facilitate short-term dimensional recovery through greater matrix compliance, but does not necessarily improve long-term thickness retention. The recovery response therefore exhibits an intermediate optimum rather than a monotonic advantage with increasing plasticization. The influence of microsphere loading is most clearly resolved in Figure 7c. Under constant 30P matrix composition and HD conditions, increasing microsphere content from B1 to B2 improved the recovery before Day 2 from approximately 97.58% to complete recovery, while the final retained thickness increased from 93.10% to 96.49%. Increasing the microsphere loading further to B3 reduced the final value to 95.25%. The B2 formulation therefore provided the most favorable balance between recoverable deformation and cumulative dimensional retention within the 30P-HD series. This non-monotonic dependence is consistent with the broader formulation behavior observed in the density and tensile results, where increasing microsphere loading beyond an intermediate level did not necessarily produce a further improvement in structural performance.

Overall, the thickness-recovery results distinguish short-term recovery from long-term dimensional retention. Highly plasticized formulations such as 30P can recover rapidly after unloading, but this does not translate directly into minimum cumulative set after repeated loading. Conversely, intermediate matrix resistance and microsphere loading can provide more favorable long-term retention. This distinction is important for the interpretation of the subsequent cyclic parameters: the thickness measurements in Figure 7 describe macroscopic dimensional recovery over extended resting periods, whereas the residual strain, hysteresis, and energy-dissipation parameters examined in the following figures quantify the response within and between individual loading cycles. Accordingly, recovery and damping should be treated as separate performance criteria rather than as interchangeable measures of cyclic performance.

**Figure 8:**
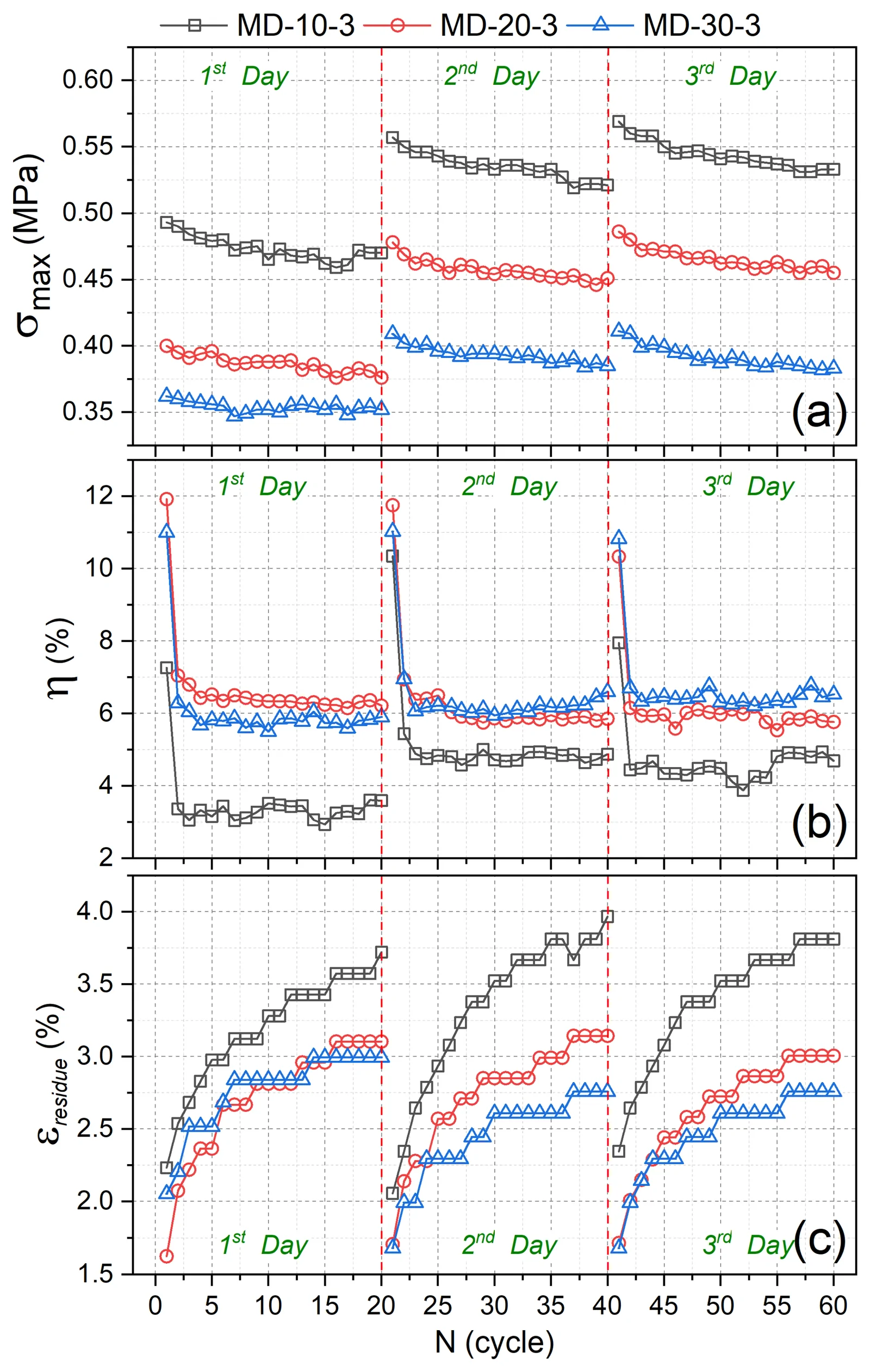
Cyclic response of B3-MD formulations with different processing-oil contents over three consecutive test days: (**a**) σmax, (**b**) η, and (**c**) εres, as a function of cycle number.

**Figure 9:**
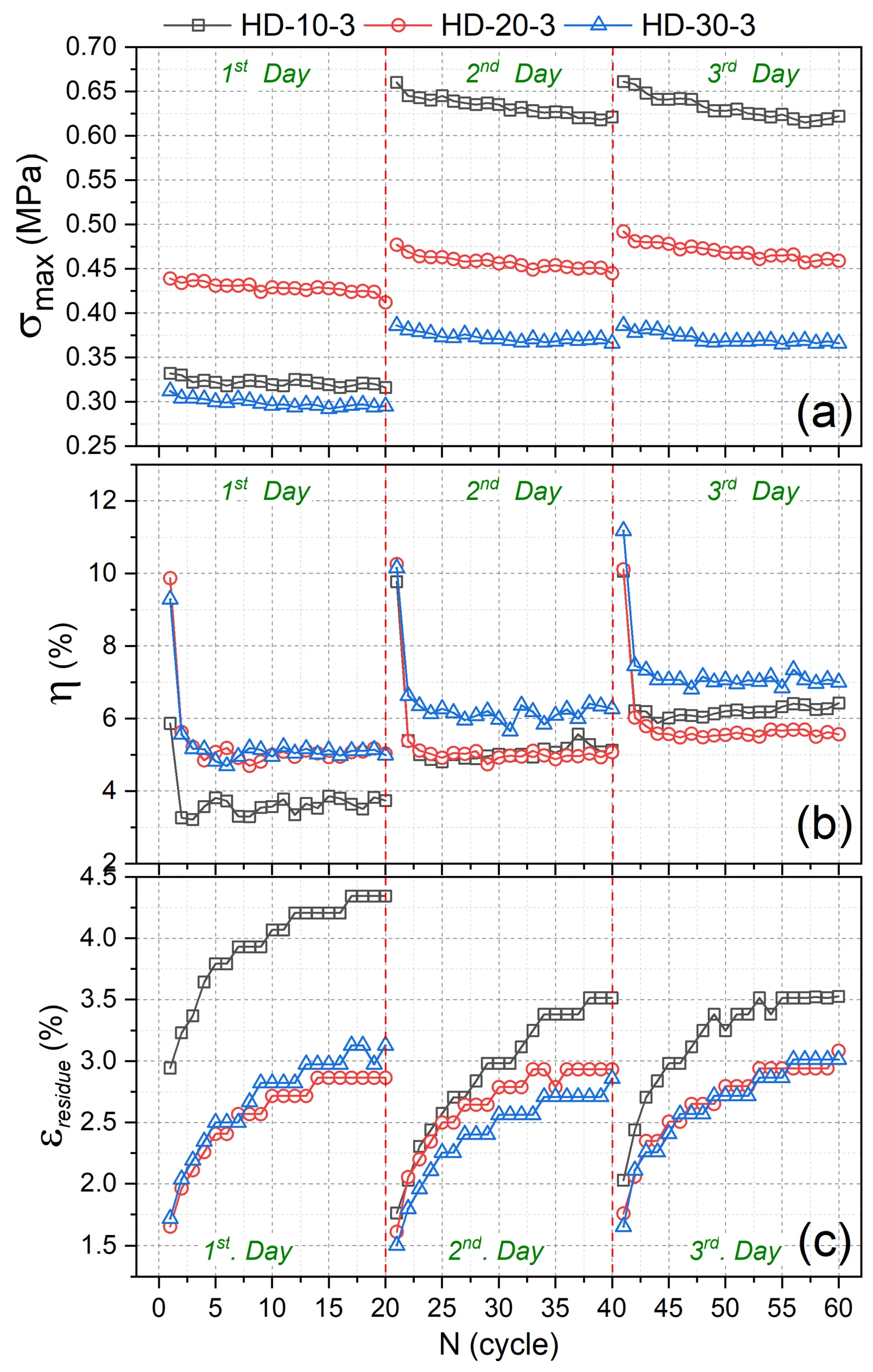
Cyclic response of B3-HD formulations with different processing-oil contents over three consecutive test days: (**a**) σmax, (**b**) η, and (**c**) εres, as a function of cycle number.

For the B3 formulations that achieved the nominal target density, the cyclic response was further evaluated through the evolution of σmax, η, and εres over three consecutive test days. These parameters describe complementary aspects of the cyclic behavior: load-bearing capacity, energy dissipation, and cycle-resolved residual deformation, respectively. Unlike the long-term thickness recovery presented in Figure 7, εres represents the deformation remaining at the end of individual loading cycles and therefore probes recovery over a substantially shorter time scale. The corresponding results for the MD and HD series are presented in Figure 8 and Figure 9, respectively.

For the MD series (Figure 8), σmax decreased systematically with increasing processing-oil content, maintaining the order MD-10-3 > MD-20-3 > MD-30-3 throughout the three-day test. Within each day, the response generally decreased during the first cycles and subsequently approached a comparatively stable level, indicating progressive cyclic conditioning. The η response showed the opposite formulation dependence. After the transient first-cycle peaks, the conditioned η values were approximately 3–4% for MD-10-3 and 6–7% for MD-30-3, corresponding to an almost twofold increase in energy dissipation with increasing matrix plasticization. This enhancement was accompanied by lower cycle-resolved residual deformation: toward the later cycles, εres approached approximately 4% for MD-10-3 but remained around 2.8% for MD-30-3. The MD results therefore establish a clear trade-off in which increasing processing-oil content reduces cyclic stress capacity while enhancing dissipation and deformation accommodation.

The HD series (Figure 9) exhibited the same general formulation dependence after conditioning, but the first test day showed a distinctive response for HD-10-3. During Day 1, this formulation exhibited both an unusually low σmax of approximately 0.32 MPa and the highest εres, reaching about 4.3%, whereas σmax increased to approximately 0.65 MPa at the beginning of Day 2 and remained at a similarly high level on Day 3. The simultaneous occurrence of low stress capacity and high residual deformation during the first loading sequence is consistent with an initial bedding-in or seating effect of the stacked specimens, in which interlayer gaps and local surface irregularities are progressively accommodated during the first series of cycles. This effect was pronounced only for HD-10-3, the stiffest of the foamed formulations (E = 14.72 MPa). This may be associated with its higher stiffness: under the low contact preload, the softer, higher-oil discs are expected to conform to each other at the interfaces, whereas the stiffer HD-10-3 discs may retain interlayer gaps that are closed only during the first loading cycles. The subsequent Day 2–3 response is therefore more representative for formulation comparison within the conditioned state. Under these conditions, σmax again followed the expected decrease with increasing oil content, with HD-10-3 carrying the highest cyclic stress and HD-30-3 the lowest. The damping response of the HD series also evolved with conditioning. HD-10-3 exhibited the lowest η during Day 1, whereas the difference between the formulations narrowed during the following days. Nevertheless, HD-30-3 maintained the highest sustained η during the conditioned cycles, particularly on Day 3, while the higher-oil formulations generally accumulated less εres than HD-10-3. Thus, the HD data support the same strength–damping trade-off observed for MD, although the pronounced first-day conditioning of HD-10-3 demonstrates that cyclic history can substantially influence the measured response before a stable deformation state is established.

Taken together, the MD and HD results show that matrix resistance controls different aspects of cyclic performance in opposing directions. The 10P formulations provide the greatest load-bearing capacity after conditioning, whereas increasing processing-oil content promotes hysteretic energy dissipation and generally limits cycle-resolved residual deformation. The 30P formulations should therefore not be interpreted as general recovery optima; their clearest functional advantage lies in sustained damping under repeated loading, while intermediate formulations can provide more favorable long-term dimensional retention as shown in Figure 7. This separation between cyclic strength, dimensional recovery, residual deformation, and energy dissipation reinforces the need to define formulation performance according to the targeted mechanical function rather than through a single optimum composition [5,6].

**Figure 10:**
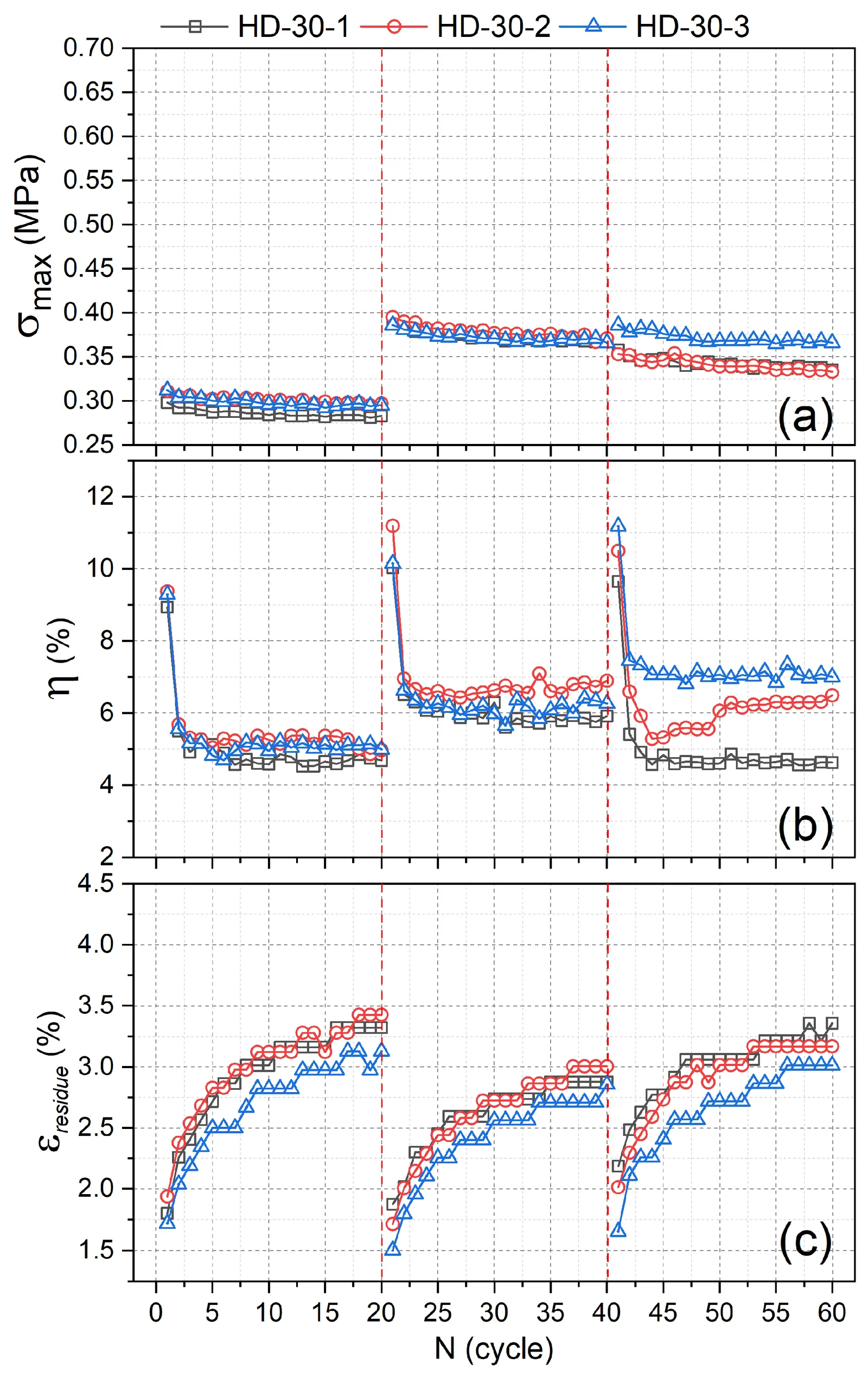
Cyclic response of 30P-HD formulations with different expandable-microsphere loadings over three consecutive test days: (**a**) σmax, (**b**) η, and (**c**) εres, as a function of cycle number.

The effect of expandable-microsphere loading on the cyclic behavior was isolated within the 30P-HD series in Figure 10, where the matrix composition and density condition were kept constant while the microsphere content varied from 1 to 3 phr. During Day 1, the three formulations exhibited very similar σmax values. A clearer separation emerged after conditioning: on Day 2, HD-30-2 maintained the highest σmax, whereas on Day 3 the three formulations initially started at comparable levels but HD-30-3 showed the best stress retention during the later cycles as the B1 and B2 responses progressively decreased. Thus, the effect of microsphere loading on cyclic load-bearing capacity was non-monotonic, with the intermediate B2 loading remaining favorable in the conditioned state and the B3 formulation becoming advantageous primarily in terms of late-cycle stress retention.

The loading dependence was more distinct in the damping and residual-strain responses. Following the transient η peaks at the beginning of each test day, HD-30-3 generally maintained the highest conditioned damping, particularly on Day 3, where η remained close to 7% compared with approximately 4.5–5% for HD-30-1. At the same time, HD-30-3 consistently remained at the lower end of the εres range, reaching approximately 3.0% by the end of Day 3, compared with values above 3.2% for the lower-loading formulations. These results indicate that the higher microsphere content favors sustained hysteretic dissipation and limits cycle-resolved residual deformation, even though it does not provide a universal advantage in cyclic stress capacity. The distinction between B2 and B3 is therefore important. The intermediate B2 loading was already identified as favorable in several previous metrics, including long-term thickness retention in Figure 7 and selected quasi-static mechanical responses, and it also provided the highest σmax during Day 2 in Figure 10. Increasing the microsphere content further to B3 did not improve every performance parameter but instead shifted the balance toward higher damping and lower cycle-resolved residual strain. This behavior is consistent with the broader non-monotonic formulation response observed throughout the study, in which increasing microsphere loading beyond an intermediate level does not necessarily improve structural performance uniformly. Accordingly, Figure 10 reinforces the conclusion that microsphere loading should not be optimized using a single criterion. Within the 30P-HD system, B2 provides a more favorable balance for dimensional retention and parts of the load-bearing response, whereas B3 becomes more advantageous when sustained energy dissipation and reduced cycle-resolved residual deformation are prioritized. This final comparison further supports the central conclusion that the optimum cellular formulation depends on the targeted mechanical function rather than on a single universal composition.

## 4. Conclusions

This study demonstrates that matrix rheological resistance plays a central role in governing the expansion and cellular retention behavior of expandable-microsphere-filled SBS foams. Increasing processing-oil content reduced the elastic and flow resistance of the matrix by approximately 2–3-fold across the investigated formulation range, facilitating microsphere expansion under moderate density-reduction requirements. This benefit was not unlimited: under the most demanding LD condition, further softening no longer improved retained expansion and instead produced the clearest signs of structural instability. The resulting behavior indicates a non-monotonic balance between matrix softening, microsphere expansion, and cellular retention rather than a continuously beneficial effect of decreasing matrix resistance.

The structural and functional results further demonstrate that density reduction, pore development, and mechanical performance cannot be optimized independently. Dielectric measurements provided a bulk-sensitive complement to two-dimensional optical microscopy by indicating that larger average pores at comparable density did not necessarily correspond to a higher total void fraction. The smooth density-dependent ε′ response of the stable formulations was also consistent with a predominantly discrete, closed-cell architecture. At the same time, MD-30-3 exhibited the largest mean pore size, whereas the highest mean quasi-static toughness was obtained in the 10P-HD region, with HD-10-3 reaching 11.50 MJ m^−3^.

Cyclic testing introduced a further function-dependent trade-off. Increasing processing-oil content reduced cyclic load-bearing capacity but increased conditioned damping, with η rising from approximately 3–4% for MD-10-3 to 6–7% for MD-30-3, while generally reducing cycle-resolved residual strain. Long-term dimensional retention, however, favored intermediate formulations. A similar non-monotonic dependence was observed for microsphere loading: B2 provided the more favorable long-term thickness retention, whereas B3 became more advantageous for sustained damping and lower cycle-resolved residual deformation. Thus, the formulation producing the largest pores, highest tensile strength, best dimensional retention, or strongest damping was not the same.

Overall, the main contribution of this work is not the identification of a single optimum SBS foam composition, but the demonstration of a rheology-guided design strategy in which matrix resistance can be tuned to control cellular development, dielectric response, quasi-static mechanics, and cyclic functionality along a common formulation axis. Direct three-dimensional cell characterization and isolation of the individual microsphere-shell contribution were beyond the scope of the present study and remain appropriate targets for future work. Within these limits, the results provide a function-specific framework for designing lightweight SBS foams beyond density reduction alone.

## Figures and Tables

**Table 1:** Tensile properties of SBS-based formulations with different processing-oil and expandable-microsphere contents. Values are reported as mean ± standard deviation (*n* ≥ 4).

Sample	Elastic Modulus, E (MPa)	Maximum Stress (MPa)	Strain at Break (%)	Toughness (MJ m^−3^)
SBS-10	23.23 (±2.12)	1.761 (±0.245)	449 (±105)	7.17 (±0.58)
LD-10-1	7.19 (±1.02)	1.504 (±0.226)	263 (±12)	3.42 (±0.28)
LD-10-2	6.41 (±0.42)	1.508 (±0.128)	362 (±40)	4.72 (±0.31)
LD-10-3	8.03 (±0.59)	1.703 (±0.221)	489 (±47)	7.46 (±0.47)
MD-10-1	12.56 (±1.13)	2.387 (±0.353)	456 (±38)	9.49 (±1.05)
MD-10-2	12.85 (±1.21)	2.061 (±0.235)	426 (±55)	7.73 (±0.62)
MD-10-3	13.27 (±1.65)	2.105 (±0.185)	469 (±18)	8.63 (±0.82)
HD-10-1	11.33 (±1.23)	2.303 (±0.285)	510 (±48)	10.02 (±0.92)
HD-10-2	13.04 (±0.95)	2.283 (±0.211)	502 (±55)	10.21 (±1.12)
HD-10-3	14.72 (±1.05)	2.531 (±0.185)	520 (±26)	11.50 (±0.99)
SBS-20	5.66 (±1.05)	1.744 (±0.353)	530 (±23)	5.91 (±1.78)
LD-20-1	4.24 (±0.45)	1.006 (±0.355)	119 (±12)	0.98 (±0.11)
LD-20-2	5.70 (±0.38)	1.293 (±0.152)	114 (±6)	1.16 (±0.13)
LD-20-3	6.08 (±0.41)	1.241 (±0.090)	85 (±5)	0.83 (±0.06)
MD-20-1	6.03 (±0.27)	1.393 (±0.122)	254 (±11)	3.00 (±0.12)
MD-20-2	5.37 (±0.57)	1.379 (±0.127)	298 (±23)	3.47 (±0.35)
MD-20-3	4.64 (±0.45)	1.798 (±0.192)	289 (±18)	4.33 (±0.35)
HD-20-1	6.04 (±0.11)	2.009 (±0.103)	511 (±16)	8.76 (±0.83)
HD-20-2	6.42 (±0.18)	2.386 (±0.241)	445 (±42)	8.72 (±0.85)
HD-20-3	5.81 (±0.26)	2.417 (±0.175)	507 (±38)	9.65 (±0.84)
SBS-30	3.94 (±1.10)	1.053 (±0.201)	118 (±18)	0.96 (±0.48)
LD-30-1	2.69 (±0.15)	0.861 (±0.081)	113 (±12)	0.75 (±0.04)
LD-30-2	3.80 (±0.27)	1.072 (±0.127)	131 (±4)	1.10 (±0.13)
LD-30-3	3.02 (±0.27)	0.942 (±0.111)	79 (±11)	0.54 (±0.13)
MD-30-1	4.86 (±0.42)	1.383 (±0.087)	162 (±14)	1.77 (±0.21)
MD-30-2	3.42 (±0.38)	1.295 (±0.075)	212 (±25)	2.18 (±0.32)
MD-30-3	5.96 (±0.47)	1.668 (±0.145)	113 (±13)	1.40 (±0.13)
HD-30-1	3.48 (±0.11)	1.721 (±0.215)	298 (±30)	3.92 (±0.41)
HD-30-2	4.92 (±0.71)	1.792 (±0.121)	266 (±36)	3.94 (±0.43)
HD-30-3	4.01 (±0.45)	1.741 (±0.178)	259 (±15)	3.59 (±0.37)

## Data Availability

The data supporting the findings of this study are available from the corresponding author upon reasonable request.

## References

[1] Aimar C., Orgéas L., du Roscoat S.R., Bailly L., Sentis D.F. (2023). Fatigue Mechanisms of a Closed Cell Elastomeric Foam: A Mechanical and Microstructural Study Using Ex Situ X-ray Microtomography. Polym. Test..

[2] Zhai W., Jiang J., Park C.B. (2022). A Review on Physical Foaming of Thermoplastic and Vulcanized Elastomers. Polym. Rev..

[3] Zhan H., Li X., Yuan C., Qian J., Mi H.-Y., Dong B., Liu C., Shen C. (2024). Lightweight and Tough Polymer Foam with Defect-Free Surface and Bimodal Cell Prepared by Thermally Expandable Microspheres Injection Molding. Polymer.

[4] Zhang R.-Z., Chen J., Huang M.-W., Zhang J., Luo G.-Q., Wang B.-Z., Li M.-J., Shen Q., Zhang L.-M. (2019). Synthesis and Compressive Response of Microcellular Foams Fabricated from Thermally Expandable Microspheres. Chin. J. Polym. Sci..

[5] He Q., Qian W., Qiu X., Zhang L. (2025). Long-Chain Cross-Linking for Enhanced Mechanical Damping of Poly(styrene-butadiene-styrene) Elastomer. ACS Appl. Polym. Mater..

[6] Zhou X., Wu M., Gao P., Li X., Ma W., Wang L., Zheng W. (2024). High-Expansion Injection-Molded Thermoplastic Polyester Elastomer Foam with Oriented Cellular Structure and Anisotropic Compressive Properties. ACS Appl. Polym. Mater..

[7] Sharudin R.W.B., Ohshima M. (2013). Preparation of Microcellular Thermoplastic Elastomer Foams from Polystyrene-b-ethylene-butylene-b-polystyrene (SEBS) and Their Blends with Polystyrene. J. Appl. Polym. Sci..

[8] Canevarolo S.V., Mattoso L.H.C. (1990). Preferential Plasticization of SBS Triblock Copolymer. Br. Polym. J..

[9] Flosenzier L.S., Torkelson J.M. (1992). Influence of Molecular Weight and Composition on the Morphology and Mechanical Properties of SBS-Polystyrene-Mineral Oil Blends. Macromolecules.

[10] Huy T.A., Hai L.H., Adhikari R., Weidisch R., Michler G.H., Knoll K. (2003). Influence of Interfacial Structure on Morphology and Deformation Behavior of SBS Block Copolymers. Polymer.

[11] Sengupta P., Noordermeer J.W.M. (2004). Effects of Composition and Processing Conditions on Morphology and Properties of Thermoplastic Elastomer Blends of SEBS-PP-Oil and Dynamically Vulcanized EPDM-PP-Oil. J. Elastomers Plast..

[12] Huang T., Xu P., Wang D., Li W., Wei R., Ji Q., Lin H., Shen J. (2025). High-Temperature-Resistant Organic–Inorganic Hybrid Microspheres with Superior Thermal Expansion Properties. ACS Appl. Polym. Mater..

[13] Seo S.E., Kwon Y., Dolinski N.D., Sample C.S., Self J.L., Bates C.M., Valentine M.T., Hawker C.J. (2021). Three-Dimensional Photochemical Printing of Thermally Activated Polymer Foams. ACS Appl. Polym. Mater..

[14] Chen G.-S., Li X.-K., Yang W.-C. (2025). Preparation of Microcellular High-Density Polyethylene with Thermal Expandable Microspheres. Polymers.

[15] Jonsson M., Nordin O., Kron A.L., Malmström E. (2010). Influence of Crosslinking on the Characteristics of Thermally Expandable Microspheres Expanding at High Temperature. J. Appl. Polym. Sci..

[16] Kawaguchi Y., Oishi T. (2004). Synthesis and Properties of Thermoplastic Expandable Microspheres: The Relation between Crosslinking Density and Expandable Property. J. Appl. Polym. Sci..

[17] Kmetty Á., Litauszki K. (2020). Development of Poly(Lactide Acid) Foams with Thermally Expandable Microspheres. Polymers.

[18] Wang L., Yang X., Zhang J., Zhang C., He L. (2014). The Compressive Properties of Expandable Microspheres/Epoxy Foams. Compos. Part B Eng..

[19] Lee H., Gupta A., Mekonnen T.H. (2023). Fabrication of Triblock Elastomer Foams for Oil Absorption Applications: Effects of Crosslinking, Composition, and Rheology Factors. ACS Appl. Polym. Mater..

[20] Chen J., Yang L., Chen D., Mai Q., Wang M., Wu L., Kong P. (2021). Cell Structure and Mechanical Properties of Microcellular PLA Foams Prepared via Autoclave Constrained Foaming. Cell. Polym..

[21] Moradian M., Azdast T., Doniavi A. (2020). Investigating the Effect of Foam Properties on the Attenuation of Coaxial Cables with Foamed Polyethylene Dielectric. Polym. Adv. Technol..

[22] Bor J., Lafond O., Merlet H., Le Bars P., Himdi M. (2014). Technological Process to Control the Foam Dielectric Constant: Application to Microwave Components and Antennas. IEEE Trans. Compon. Packag. Manuf. Technol..

[23] Chand N., Sharma J. (2012). Influence of Porosity on Resistivity of Polypropylene Foams. J. Cell. Plast..

[24] Petrossian G., Hohimer C.J., Ameli A. (2019). Highly-Loaded Thermoplastic Polyurethane/Lead Zirconate Titanate Composite Foams with Low Permittivity Fabricated Using Expandable Microspheres. Polymers.

[25] Zhao B., Zhao C., Wang C., Park C.B. (2018). Poly(vinylidene fluoride) Foams: A Promising Low-k Dielectric and Heat-Insulating Material. J. Mater. Chem. C.

[26] Zhao J., Wang Z., Nie H. (2022). High-Performance Poly(aryl ether ketone) Foams with Low Dielectric Constant and Dielectric Loss. ACS Appl. Polym. Mater..

[27] (2025). Standard Test Methods for Rubber Property—Compression Set.

[28] Polizzi S., Stribeck N., Zachmann H.G., Bordeianu R. (1988). Structure and properties of oil extended styrene butadiene block copolymers. Polym. Compos..

